# Factors Associated with Immunization Status Among Myanmar Migrant Children in Northern Thailand: A Community-Based Cross-Sectional Study

**DOI:** 10.3390/medsci14040452

**Published:** 2026-08-02

**Authors:** Shin Moe Oo, Pamornsri Inchon, Peeradone Srichan, Kriengkrai Prasert

**Affiliations:** 1 Public Health Department, School of Health Science, Mae Fah Luang University, Mueang, Chiang Rai 57100, Thailand; 6751804004@lamduan.mfu.ac.th (S.M.O.); peeradone.sri@mfu.ac.th (P.S.); 2 Faculty of Public Health, Kasetsart University, Chalermphrakiat Sakon Nakhon Campus, Mueang, Sakon Nakhon 47000, Thailand; kriengkrai.pra@ku.ac.th; 3 Clinical Research Center, Nakhon Phanom Hospital, Mueang, Nakhon Phanom 48000, Thailand

**Keywords:** immunization, coverage, children, Myanmar, migrants, Northern, Thailand

## Abstract

**Background**: Children of Myanmar migrants in Thailand have lower immunization coverage compared to Thai counterparts, leaving them at risk of vaccine preventable diseases. Evidence remains limited regarding immunization among Myanmar children in Chiang Rai Province, an important migration corridor between Northern Thailand and Myanmar. **Objective**: this study aimed to estimate the immunization status and identify its associated factors among children of Myanmar migrants in Chiang Rai province, Northern Thailand. **Methods**: A cross-sectional study was conducted among Myanmar migrant caregivers and their children aged under 7 years, who are residing in Chiang Rai Province, using a validated questionnaire by face-to-face interview. Descriptive statistics was used to summarize participant characteristics, while chi-square tests and binary logistic regressions were used to identify associated factors with statistical significance set at α< 0.05. **Results**: Among 423 children, 91 children (21.5%) were found as having incomplete immunization and six factors were significantly associated with it, including lacking Thai birth registration (aOR = 9.52; 95% CI: 3.74–24.27), lacking child’s health insurance (aOR = 14.03; 95% CI: 6.45–30.54), history of return migration (aOR = 19.05; 95% CI: 8.48–44.13), negative peer practices (aOR = 8.09, 95% CI: 1.47–44.54), time barrier (aOR = 4.09, 95% CI: 1.20–13.94) and financial barrier (aOR = 7.07, 95% CI: 1.67–29.91). **Conclusions**: A moderate prevalence of incomplete immunization was strongly associated with factors across the levels of the modified socio-ecological model: intrapersonal, interpersonal, and institutional. Interventions addressing legal access, health insurance, community outreach, healthcare system responsiveness and cross-border continuity of care are recommended.

## 1. Introduction

Immunization prevents 3.5 million to 5 million deaths every year [1]. It not only protects against infectious diseases but also prevents physical and intellectual disabilities among children [2]. In 2023, the WHO reported that around 14.5 million children (18%) out of a total 128 million children globally missed out on any vaccination, known as zero-dose children [3]. According to UNICEF, nearly half of un- and under-vaccinated children are from countries affected by conflicts and migrated across borders into neighboring nations [4]. Globally, migrant and refugee children in Europe experience lower immunization coverage than host populations because of various individual and structural barriers [5]. Despite the diverse contexts, studies in Sweden, India, and conflict-affected countries collectively reported that migrant and displaced children face immunization gaps due to population mobility, inadequate vaccination documentation, language differences, fear of accessing healthcare services, and disruption of health systems [6,7,8]. The failure to vaccinate these children not only threatens their lives but also poses a potential threat to national and global health security since vaccine-preventable disease outbreaks can spread quickly, transcending borders and leading to larger-scale public health crises [4].

In Southeast Asia (SEA), high-income countries such as Singapore, Malaysia, and Brunei Darussalam achieve immunization coverage rates above 90%, whilst low- and middle-income countries (LMICs) face significant challenges due to socio-economic disparities, healthcare access barriers, conflicts, displacement, and migration [3]. For example, Myanmar’s EPI has been severely disrupted after the COVID-19 pandemic and political conflicts, with EPI coverage for many essential vaccines dropping below 50% in 2021 [9]. In contrast, Thailand’s EPI has high national coverage, supported by strong health infrastructure and primary healthcare networks and covering 13 vaccine-preventable diseases including BCG, DTP, polio, hepatitis B, measles, MMR, Japanese encephalitis, etc., [10]. Despite Thailand’s strong coverage, outbreaks of diseases such as measles and pertussis still occur, particularly in border areas where there are high concentrations of migrant populations [11,12]. Thailand serves as a cross-border migration hub for many working-age groups and their accompanying dependents. As of 2023, it hosts approximately 4.9 million migrants, primarily constituting Myanmar nationalities for nearly 60% of this figure [13]. From 2022 onwards, Chiang Rai has become an increasingly popular destination for people from Myanmar due to several push and pull factors such as geographical proximity, increasing labor market demand, and pre-existing migrant networks [14]. There are about 17,000 registered Myanmar migrant workers residing in the Chiang Rai province [15]. Furthermore, the 2021 military coup has exacerbated parents’ concerns regarding their children’s safety, education, and healthcare. These situations urge many Myanmar families to cross borders into Thailand with their child dependents [16].

Migrant populations often face structural and systemic barriers, which can hinder timely and complete vaccination for children [17]. Studies have consistently revealed that Myanmar children in Thailand have lower immunization coverage compared to their Thai-born colleagues [18,19,20]. In Thailand, access to immunization services is influenced by the individual’s legal status and enrollment of children onto health insurance schemes. Documented migrant workers can purchase health insurance for their children from the Ministry of Public Health’s Migrant Health Insurance Scheme (MHIS), at a cost of 365 baht per year for children under 7 years and 1600 baht for those who aged 7 to 14 years [21]. However, undocumented migrants and their children are often excluded from such schemes due to legal and financial barriers [22]. Although alternative coverage is available through non-governmental initiatives such as the Migrant Fund (M-Fund), the uptake remains limited due to awareness gaps, language barriers, and mobility [22]. In addition, misinformation and vaccine hesitancy, distance to immunization service, and prioritization of work over childcare further contribute to lower immunization rates among this population [20]. Therefore, understanding immunization coverage and its determinants among children of Myanmar migrants is essential for strengthening equitable immunization services in border areas with high migrant density.

Previous studies in Thailand primarily focused on children aged under two years and were conducted in border and economic zones like Tak province and the Bangkok Metropolitan Area (BMA), with the time gap of more than a decade [18,19,20]. At present, there has been little attention to the broader age group of migrant children in an emerging migrant destination like Chiang Rai Province. Furthermore, the existing literature suggests that childhood immunization among migrants is influenced by a range of social and healthcare system factors beyond individual characteristics alone [23,24,25]. Hence, guided by the Socio-Ecological Model (SEM) [26], this study aims to assess the immunization status and its associated factors of children (age under 7 years) of Myanmar migrants. The findings are expected to support policymakers, public health practitioners, and healthcare providers in designing targeted interventions to address specific barriers for the Myanmar migrant population. Ultimately, the study may contribute to strengthening immunization programs and reducing health disparities among migrant children in Thailand.

## 2. Materials and Methods

### 2.1. Study Design and Setting

**Figure 1:**
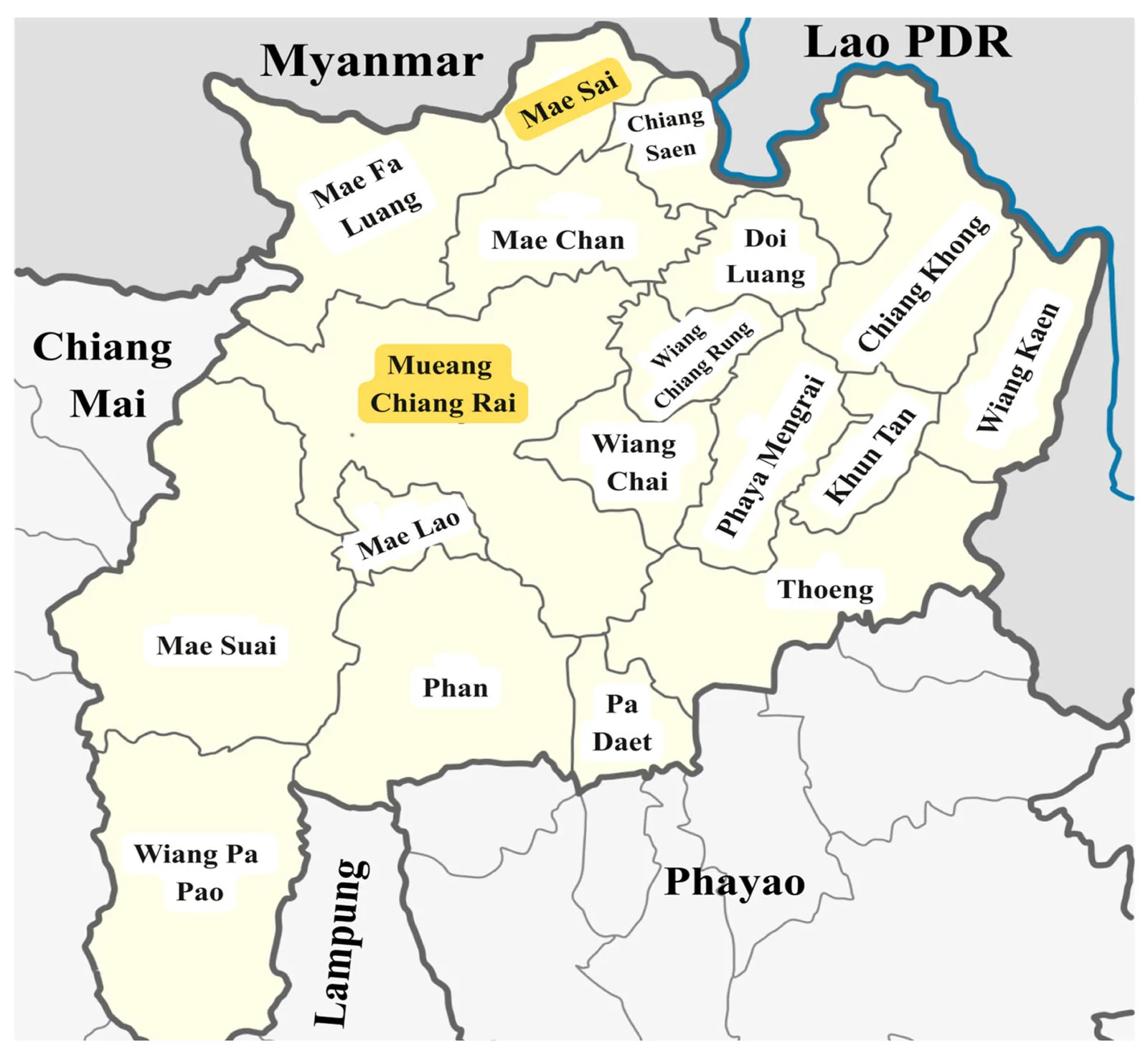
Map of Chiang Rai province showing 18 districts [28].

A community-based cross-sectional study was conducted from October to December 2025 in the Mueang Chiang Rai and Mae Sai Districts of Chiang Rai province (Figure 1), where a large population of Myanmar migrants reside [15,27].

### 2.2. Study Population, Sampling and Eligibility Criteria

The sample size was calculated using Cochran’s formula based on a prevalence estimate of 56.7% from a previous study [18]. After adding an additional 20% for potential incompleteness and withdrawals, the final sample size was 430. In this study, the study participants were Myanmar migrant caregivers (mother, father, grandparents, etc.) and their children who were the subjects of interest and the unit of analysis. The eligibility criteria were Myanmar migrant caregivers of 15 years and above with children aged under 7 years who are residing in Chiang Rai Province at the time of the study. Only caregivers who were able to communicate in the Burmese (Myanmar) language and provided informed consent to participate in the study were included. A community-based referral (snowball) sampling was employed due to the hard-to-reach nature of the migrant population, particularly undocumented migrant workers. Participants were recruited from multiple entry points such as civil society organizations (CSOs), local non-government organizations (NGOs), and migrant health volunteers (MHVs).

### 2.3. Measurement Tools and Development

A structured and validated questionnaire was used to assess the immunization status and its associated factors. The questionnaire consists of six main parts; (1) socio-demographic characteristics, (2) immunization status, (3) knowledge, (4) attitude, (5) interpersonal drivers and (6) institutional drivers of immunization.

#### 2.3.1. Independent Variables

The independent variables, based on the modified socioecological model were captured by five domains. First, the socio-demographic part included general characteristics of the child and caregiver, legal status, health insurance, return migration history and planned return migration. Caregiver and child sex were coded as male or female. Thai birth registration was categorized as “yes” or “no”, while health insurance status was classified as insured and uninsured. Return migration history was defined as whether the child had returned to Myanmar (yes or no), while planned return migration was categorized according to whether the caregiver intended to send the child back to Myanmar in the future (yes or no). Second, caregiver’s knowledge regarding childhood immunization was assessed by the questions adopted from the previous study and categorized as “poor”, “moderate”, or “good” [18]. Third, the caregiver’s attitudes toward childhood immunization were assessed using the Parental Attitudes about Childhood Vaccines (PACV) questionnaire and classified as “positive” or “negative” [29]. Fourth, the interpersonal-level factors included peer practice and social influences related to immunization. Finally, the institutional-level factors contained the accessibility of immunization services and barriers such as language, transportation, financial, time and documentation.

Interpersonal and institutional questions were adopted both from reviews of the relevant literature and the WHO’s Behavioral and Social Drivers of Immunization (BeSD) framework, using multiple choice and dichotomous (yes or no) scales [23]. Apart from knowledge section, all other questionnaires were translated with a forward–backward method into a Myanmar version by experienced bilingual translators. Content validity was assessed by three experts from the fields of public health, and pediatric and global health using the IOC scale. Items with scores between 0.50 and 0.70 were revised according to the expert’s feedback, while items with scores > 0.70 were retained. Reliability of the questionnaire was pilot-tested among 30 Myanmar migrant caregivers with characteristics similar to the study population. Following revision, all scales demonstrated acceptable internal consistency with Cronbach’s alpha > 0.70. The full questionnaire is provided in the Supplementary Materials.

#### 2.3.2. Dependent Variable

The immunization status was verified using the Thai maternal and child health record “pink book” available at the time of the study and categorized as complete or incomplete as follows:

- Complete immunization: defined as receiving all age-appropriate vaccines according to the Thai national EPI schedule, as presented in Table 1.
- Incomplete immunization: referred to missing at least one recommended vaccine dose according to the child’s age. Children without a pink book or without sufficient documented vaccination records to verify completion of the recommended schedule also fall under this category. Caregiver recall alone was not used to determine immunization status.

### 2.4. Ethical Consideration

Ethical approval was received from the Mae Fah Luang University Ethics Committee on Human Research (EC 25133-18, COA: 202/2025) in October 2025.

### 2.5. Data Collection

After receiving ethical approval, participants meeting the eligibility criteria were recruited by coordinating with local field officers and migrant community leaders. Before the questionnaire phase, each caregiver was provided with an explanation of the study’s purpose and benefits, and then asked to sign the consent form assuring that the participation in this study was completely voluntary. A structured and validated questionnaire was administered by the researcher in face-to-face interviews, conducting them individually at their residence or community assembly areas according to their availability. To ensure the confidentiality and anonymity of participants, all collected data were carefully entered and encoded in Microsoft Excel.

### 2.6. Statistical Analysis

Data were analyzed using computer software—IBM SPSS Statistics for Mac, Version 30.0 (IBM Corp., Armonk, NY, USA). Descriptive statistics were presented as frequencies and percentages for categorical variables. The normality of continuous variables was assessed using the Shapiro–Wilk test. Variables that were not normally distributed were summarized using the median and interquartile range (IQR), whereas normally distributed variables were presented as mean and standard deviation (SD). Categorical variables were compared using Pearson’s chi-square test or Fisher’s exact test if the expected frequency in any cell of the contingency table was less than five. Variable selection for multivariable logistic regression was guided by the modified socio-ecological framework, epidemiological relevance and the results of the univariable logistic regression. Independent variables were grouped into intrapersonal, interpersonal, and institutional domains according to the framework. Variables with *p* < 0.20 in univariable analysis were initially considered for multivariable analysis. Collinearity among candidate variables was assessed using variance inflation factors (VIFs), with VIF > 5 or tolerance < 0.2 considered highly collinear and excluded based on theoretical relevance. The remaining variables were then entered into the multivariable logistic regression model. The final multivariable model was fitted using the enter method, in which all selected variables were entered simultaneously. Both crude odds ratios (cORs) and adjusted odds ratios (aORs) with 95% confidence intervals (CIs) were reported, and statistical significance was defined as *p* < 0.05.

## 3. Results

### 3.1. Immunization Status of Myanmar Migrant Children

A total of 430 children were recruited. After data collection, seven questionnaires were excluded because of incomplete responses. Therefore, 423 children were included in the final analysis. Among the total 423 children, 332 children (78.5%) were completely immunized according to the age-appropriate Thai EPI schedule, while 91 children (21.5%) had incomplete immunization.

### 3.2. Bivariate Analysis

Bivariate analyses using chi-square tests examined the association between immunization status and factors at the intrapersonal, interpersonal, and institutional levels as follows.

#### 3.2.1. Association Between Intrapersonal Factors and Immunization Status

Regarding the intrapersonal factors, caregiver-related characteristics including relationship to child, occupation, duration in Thailand, legal status, attitude, health insurance were found to be significantly associated with the immunization status of children. Child-related factors such as age, birth country, and birth setting were also significant. Moreover, children without birth registration or health insurance in Thailand, and those with a history of return migration or a future plan for return migration to Myanmar were significantly associated with the immunization status. Detailed information can be found in Table 2.

#### 3.2.2. Association Between Interpersonal Factors and Immunization Status

At the interpersonal level, negative peer practices regarding immunization, family support, support from religious and community leaders, recommendations from Thai individuals, and information obtained from migrant community members were significantly associated with immunization status. Other interpersonal factors, including autonomy and information from family or friends were not significantly associated. Details can be found in Table 3.

#### 3.2.3. Association Between Institutional Factors and Immunization Status

Institutional or health system-related factors including the place, travel distance, cost and time taken for one visit of immunization were found to be significantly associated with the child’s immunization status. Regarding health information about immunization, information about functions, adverse events following immunization (AEFI) and vaccine schedule were significant. In addition, the perceived barriers to immunization was also significant. More specifically, time barriers, financial barriers, documentation and transportation barriers were significantly associated with the immunization status. Further details are mentioned in Table 4.

### 3.3. Univariable and Multivariable Logistic Regression Analysis

Univariable logistic regression analysis identified 30 variables with a *p*-value < 0.20 which were considered candidate variables for inclusion in the multivariable logistic regression model. After removing variables with strong collinearity, six factors remained significantly associated with incomplete immunization in the final model of the multivariable logistic regression. Children without health insurance were 14 times more likely to have incomplete immunization compared to those who were insured (aOR = 14.03, 95% CI: 6.45–30.54). Next, children without Thai birth registration were about 9.5 times more likely to have incomplete immunization than those who had (aOR = 9.52, 95% CI: 3.74–24.27). In addition, children with a history of returning to Myanmar for more than one year were 19 times more likely to have incomplete immunization compared to those without such history (aOR = 19.35, 95% CI: 8.48–44.13). At the interpersonal level, caregivers who perceived that other parents did not regularly practice child immunization were about eight times more likely to have children with incomplete immunization than those who perceived positive peer practices (aOR = 8.09, 95% CI: 1.47–44.54). At the institutional level, caregivers with time barriers to access immunization services had a four times higher chance of having children with incomplete immunization (aOR = 4.09, 95% CI: 1.20–13.94) compared to those without such barrier. Similarly, caregivers experiencing financial difficulty had a seven times higher chance of incomplete immunization in children compared to those without such constraint (aOR = 7.07, 95% CI: 1.67–29.91). Detail information is shown in Table 5.

## 4. Discussion

This cross-sectional study revealed that 21.5% (91 out of 423) of children had incomplete immunization, which was comparatively lower than the similar studies conducted in Tak province (2011) and the Bangkok Metropolitan area (2013) [18,30]. This discrepancy may be attributed to differences in study periods and scopes, as previous studies were conducted more than a decade ago and focused on a narrower age group (under two years).

Regarding associated factors, children without health insurance had significantly higher odds of incomplete immunization compared to those covered by insurance (Table 5). This finding aligns with a study of Myanmar migrant children in a special economic zone (SEZ), Mae Sot, which reported that children with health insurance were more likely to utilize healthcare services, including immunization [22]. It is also consistent with the global evidence review from the WHO underscoring that children with health insurance coverage are more likely to receive complete immunization, as insurance reduces financial barriers and improves access to healthcare services [17]. This association can be explained by the fact that health insurance may facilitate access to health services and strengthen linkage to the formal health system [31]. Moreover, uninsured migrant families may face concerns regarding healthcare costs or documentation requirements, which may discourage healthcare utilization even for services that are officially free of charge [24].

Birth registration has been consistently identified as an important determinant of access to immunization services. A study in the Dominican Republic reported that children without birth certificates were significantly less likely to receive routine vaccines and to be fully immunized by 12 months of age [32]. Similarly, the research done on migrant children along Thai–Myanmar border area stated that children with birth certificates were more likely to have health insurance, thereby improving access to healthcare services [22]. Our findings support those previous pieces of evidence by highlighting Thai birth registration as a significant factor associated with immunization completion (Table 5). Children without birth registration had higher odds of incomplete immunization. This may reflect the role of birth registration certificates in facilitating inclusion in the formal health system, improving eligibility for health insurance, and enabling effective follow-up within routine immunization services [22].

This study also demonstrated a strong association between return migration and incomplete immunization, with children who had a history of returning to Myanmar showing significantly higher odds of incomplete immunization (Table 5). This finding is comparable with a study conducted in the Bangkok metropolitan area reporting that migrant children who frequently move or spend periods outside Thailand are at increased risk of missing scheduled vaccinations [30]. Similarly, two studies performed on immunization among children of Myanmar migrants in Mae Sot in 2010 and 2018 have shown that population mobility and unstable living conditions are major barriers to immunization [18,25]. Migration-related movement may lead to missed vaccination appointments, loss of immunization records, and reduced access to healthcare services. In addition, cross-border movement can create gaps between health systems, further limiting the continuity of immunization services. These findings highlight the importance of strengthening cross-border health coordination and implementing digital tracking systems to ensure the continuity of immunization among migrant children.

In our study, caregivers who perceived that other parents did not regularly practice childhood immunization were more likely to have incompletely immunized children compared with those reporting positive peer practices (Table 5). These findings are consistent with previous studies highlighting the influence of social norms and peer behaviors on vaccination decisions. A study conducted in the United States reported that children were 1.75 times more likely to be vaccinated when caregivers perceived that most parents within their social networks vaccinated their children [33]. In addition, the WHO Behavioural and Social Drivers (BeSD) framework identifies “social processes,” including peer influence, social norms, and community expectations as important determinants influencing vaccination uptake [23]. This association may be particularly important among migrant populations, where caregivers often rely on close peers for information, emotional support, and practical guidance in navigating healthcare services. Therefore, community-based immunization-promotion strategies may help strengthen positive peer influence and improve vaccine uptake among Myanmar migrant children.

As another significant factor, caregivers who perceived having time difficulties were more likely to have children with incomplete immunization compared to those without the barrier (Table 5). Previous studies also demonstrated that limited time availability and competing responsibilities negatively affect childhood immunization uptake. A global overview of systematic reviews on parental barriers to childhood vaccination identified lack of time, competing priorities, and difficulty attending health services as common reasons for incomplete immunization among caregivers [34]. Similarly, a cross-sectional study conducted in rural Malawi demonstrated that longer travel time and limited accessibility to health facilities were associated with lower immunization coverage among children [35]. In the context of migrant populations in Thailand, a qualitative study among Myanmar migrant families in Tak Province reported that long working hours, inflexible employment conditions, and difficulty leaving the workplace reduced caregivers’ time to bring children for vaccination services [20]. This might be because of the nature of migrant livelihoods, where caregivers often engage in informal or labor-intensive work with limited flexibility, making it difficult to attend health services during standard clinic hours [20]. In addition, long waiting times at healthcare facilities and limited clinic operating hours may further discourage caregivers from completing vaccination schedules [35]. Therefore, strengthening flexible and migrant-friendly immunization services, such as outreach vaccination programs, mobile clinics, weekend services, or community-based vaccination campaigns, are recommended to reduce time difficulty for immunization.

Finally, financial difficulty was found to be significantly associated with incomplete immunization (Table 5). This was supported by a global overview of systematic reviews on parental barriers to childhood vaccination, highlighting both direct and indirect costs, including transportation expenses, loss of daily wages, and service-related costs as important barriers affecting caregivers’ ability to complete childhood immunization schedules [34]. Likewise, studies conducted in low- and middle-income countries reported that children from economically disadvantaged households were less likely to receive complete immunization compared to those from higher socioeconomic groups, highlighting the influence of financial inequality on healthcare access [36,37]. Moreover, a qualitative study among Myanmar migrants in Tak Province, Thailand, further explained that unstable income, insecure employment, and limited social protection systems intensified financial difficulties in accessing healthcare services, including immunization [20]. Although routine childhood vaccines are provided free of charge or at minimal cost under the Thai Expanded Program on Immunization (EPI), migrant caregivers may still face substantial indirect expenses when accessing services. These include transportation costs, loss of income from missing work, and fees related to interpretation or communication support within healthcare settings [34]. For migrant families with unstable and low household income, such financial burdens may discourage timely attendance at vaccination appointments and contribute to incomplete immunization. Therefore, strengthening migrant-inclusive healthcare-support systems, reducing indirect service costs, and improving accessible communication and interpretation services should be implemented to reduce financial barriers.

## 5. Strengths and Limitations

This study has limited generalizability due to the cross-sectional design, self-reported data and the use of snowball sampling—which may have introduced selection bias as participants were primarily recruited through close community networks. Moreover, the classification of immunization status was based on documented vaccination records. Thus, some migrant children who had received vaccinations outside Thailand but lacked verifiable documentation may have been classified as incompletely immunized. This approach may potentially overestimate the prevalence of incomplete immunization. Nevertheless, it provides important context-specific evidence regarding immunization challenges among a hard-to-reach migrant population.

## 6. Conclusions

This study estimated the immunization status and identified factors associated with incomplete immunization among children (under 7 years) of Myanmar migrants in Chiang Rai Province. Approximately one in five children had incomplete status. Lack of child health insurance, absence of Thai birth registration, return migration history, negative peer practice regarding immunization, time barriers, and financial barriers were independently associated with incomplete immunization, highlighting the influence of structural, migration-related, interpersonal, and healthcare system factors. Overall, the findings emphasize that improving immunization coverage among children of Myanmar migrants requires multi-level and migrant-inclusive strategies addressing structural, social, and healthcare-system barriers. Strengthening cross-border health coordination, expanding migrant-friendly health services, improving legal access, and enhancing community-based immunization promotion may help reduce immunization inequities in border regions of Thailand. Future longitudinal and qualitative studies are recommended to further explore barriers to immunization among migrant populations and evaluate migrant-friendly immunization interventions.

## Figures and Tables

**Table 1:** Thailand EPI Schedule for Children under aged 7 years (2024) [10].

Child’s Age	Immunization Schedule by Age	Vaccines Given
Under 12 months	At birth	BCG, HB 1
	2 months	DTP-HB-Hib 1, IPV 1, Rota 1
	4 months	DTP-HB-Hib 2, IPV 2, Rota 2
	6 months	DTP-HB-Hib 3, OPV 3, Rota 3
	9 months	MMR 1
1–2 years	1 year	LAJE 1
	1 year and 6 months	DTP 4, OPV 4, MMR 2
2-Under 7 years	2 years and 6 months	LAJE 2
	4–6 years	DTP 5, OPV 5

**Table 2:** Intrapersonal factors associated with immunization status (*n* = 423).

Variables	Categories	Immunization Status				*p* Value
		Complete (*n* = 332)		Incomplete (*n* = 91)		
		*n*	%	*n*	%	
Caregiver						
Age (year)	<35 ≥35	159 173	47.9 52.1	40 51	44.0 56.0	0.554
Sex	Male Female	26 306	7.8 92.2	10 81	11.0 89.0	0.395
Relationship to child	Mother Others (father, grandparents, etc.)	301 31	90.7 9.3	75 16	82.4 17.6	0.037 *
Education	No formal education Educated	14 318	4.2 95.8	3 88	3.3 96.7	0.484
Occupation	Unemployed Employed	108 224	32.5 67.5	17 74	18.7 81.3	0.013 *
Ethnicity	Bamar Karen Others	295 14 23	88.9 4.2 6.9	84 5 2	92.3 5.5 2.2	0.213
Religion	Buddhist Christian Islam	300 22 10	90.4 6.6 3.0	84 6 1	92.3 6.6 1.1	0.760
Place of origin	Central region (Mandalay, Magway) South & west (Yangon, Rakhine, Pago) North & eastern (Shan, Karen, Mon)	250 48 34	75.3 14.5 10.2	59 21 11	64.8 23.1 12.1	0.099
Duration in Thailand (year)	<10 ≥10	117 215	35.2 64.8	52 39	57.1 42.9	<0.001 *
Legal status	Documented Undocumented	324 8	97.6 2.4	83 8	91.2 8.8	0.010 *
Health insurance	Have Do not have	254 78	76.5 23.5	46 45	50.5 49.5	<0.001 *
Family income (THB)	<9000 90,001–14,999 ≥15,000	92 112 128	27.7 33.7 38.6	29 27 35	31.9 29.7 39.4	0.665
Number of children	<1 2–3 ≥4	95 209 28	28.6 63.0 8.4	23 61 7	25.3 76.0 7.7	0.763
Knowledge	Poor Moderate High	86 198 48	25.9 59.6 14.5	32 49 10	35.2 53.8 11.0	0.194
Attitude	Positive Negative	325 7	97.9 2.1	87 4	95.6 4.4	0.002 *
Child						
Age (month)	<12 13–59 ≥60	57 137 138	17.1 41.3 41.6	4 29 58	4.4 31.9 63.7	<0.001 *
Sex	Boy Girl	189 143	56.9 43.1	52 39	57.1 42.9	1.000
Education	No schooling Preschool Primary school	148 77 107	44.6 23.2 32.2	32 26 33	35.2 28.5 36.3	0.261
Birth country	Thailand Myanmar	316 16	95.2 4.8	53 38	58.2 41.8	<0.001 *
Birth setting	Home-based Health facility	3 329	0.9 99.1	12 79	13.2 86.8	<0.001 *
Birth registration in Thailand	Have Do not have	316 16	95.2 4.8	53 38	58.2 41.8	<0.001 *
Health insurance	Have Do not have	310 22	93.4 6.6	38 53	41.8 58.2	<0.001 *
Return migration history	Yes No	14 318	4.2 95.8	42 49	46.2 53.8	<0.001 *
Planned return migration	Yes No	21 311	6.3 93.7	17 74	18.7 81.3	<0.001 *

* Significant level at α < 0.05.

**Table 3:** Interpersonal factors associated with immunization status (*n* = 423).

Variables	Categories	Immunization Status				*p* Value
		Complete (*n* = 332)		Incomplete (*n* = 91)		
		*n*	%	*n*	%	
Peer practice of immunization	Yes No	329 3	99.1 0.9	76 15	83.5 16.5	<0.001 *
Family support	Yes No	331 1	99.7 0.3	87 4	95.6 4.4	0.008 *
Religious leader support	Yes No	317 15	95.5 4.5	77 14	84.6 15.4	<0.001 *
Community leader support	Yes No	319 13	96.1 3.9	78 13	85.7 14.3	<0.001 *
Recommendation from Thai individuals	Yes No	256 76	77.1 22.9	44 47	48.4 51.6	<0.001 *
Autonomy	Yes No	330 2	99.4 0.6	89 2	97.8 2.2	0.204
Information from family/ friends	Yes No	127 205	38.3 61.7	27 64	29.7 70.3	0.142
Information from neighbors	Yes No	12 320	3.6 96.4	091	0.0 100.0	0.315
Information from migrant community	Yes No	329 3	99.1 0.9	82 9	90.1 9.9	<0.001 *
Information from social media	Yes No	8 324	2.4 97.6	3 88	3.3 96.7	0.709

* Significant level at α < 0.05.

**Table 4:** Institutional factors associated with immunization status (*n* = 423).

Variables	Categories	Immunization Status				*p* Value
		Complete (*n* = 332)		Incomplete (*n* = 91)		
		*n*	%	*n*	%	
Place of immunization	Primary health center Government hospital Others	159 166 7	47.9 50.0 2.1	36 46 9	39.6 50.5 9.9	0.004 *
Travel distance for immunization (mins)	<15 ≥15	262 70	78.9 21.1	61 30	67.0 33.0	0.025 *
Cost for one visit (THB)	<150 ≥150	259 73	78.0 22.0	50 41	54.9 45.1	<0.001 *
Time taken for one visit (hour)	<1.5 ≥1.5	189 143	56.9 43.1	35 56	38.5 61.5	0.002 *
Reminder from clinic	Yes No	32 300	9.6 90.4	3 88	3.3 96.7	0.054
Interpreter support	Yes No	41 291	12.3 87.7	18 73	19.8 80.2	0.087
Information about functions	Yes No	156 176	47.0 53.0	25 66	27.5 72.5	<0.001 *
Information about AEFIs	Yes No	277 55	83.4 16.6	52 39	57.1 42.9	<0.001 *
Information about consequences of missing	Yes No	23 309	6.9 93.1	4 87	4.4 95.6	0.475
Information about schedule	Yes No	325 7	97.9 2.1	82 9	90.1 9.9	0.002 *
Barriers for immunization	Yes No	107 225	32.2 67.8	58 33	63.7 36.3	<0.001 *
Time barrier	Yes No	14 318	4.2 95.8	10 81	11.0 89.0	<0.001 *
Financial barrier	Yes No	6 326	1.8 98.2	14 77	15.4 84.6	<0.001 *
Documentation barrier	Yes No	1 331	0.3 99.7	2 89	2.2 97.8	<0.001 *
Language barrier	Yes No	108 224	32.5 67.5	57 34	62.6 37.4	<0.001 *
Transportation barrier	Yes No	7 325	2.1 97.9	4 87	4.4 95.6	0.261

* Significant level at α < 0.05.

**Table 5:** Univariable and multivariable logistic regression analysis: factors associated with incomplete immunization status (*n* = 423).

Independent Variables	cOR	95% CI	aOR	95% CI
Intrapersonal				
Relationship to child				
Mother	1.00			
Others	2.07 *	1.08–3.98	-	-
Occupation				
Unemployed	1.00			
Employed	2.09 *	1.81–3.73	-	-
Place of origin				
Central region	1.00			
South & west	1.85 *	1.03–3.33	-	-
North & east	1.37	0.66–2.86	-	-
Duration in Thailand (year)				
<10	1.00			
≥10	2.45 **	1.53–3.93	-	-
Caregiver health insurance				
Have	1.00			
Do not have	3.19 **	1.96–5.16	-	-
Caregiver legal status				
Documented	1.00			
Undocumented	3.91 *	1.42–10.71	-	-
Child age (months)				
<12	1.00			
13–59	3.02 *	1.01–8.97	-	-
≥60	5.99 **	2.08–17.27	-	-
Birth country				
Thailand	1.00			
Myanmar	14.16 **	7.37–27.19	-	-
Birth setting				
Health facility	1.00			
Home	16.00 **	4.59–60.44	-	-
Birth registration in Thailand				
Registered	1.00		1.00	
Not registered	14.76 **	7.94–27.42	9.52 **	3.74–24.27
Child health insurance				
Have	1.00		1.00	
Do not have	19.65 **	10.78–35.83	14.03 **	6.45–30.54
Child return migration history				
No	1.00		1.00	
Yes	11.00 **	6.13–19.75	19.05 **	8.48–44.13
Planned child return migration				
No	1.00			
Yes	3.40 **	1.71–6.77	-	-
Interpersonal				
Peer practice on immunization				
Yes	1.00		1.00	
No	21.65 **	6.11–76.65	8.09 *	1.47–44.54
Family support				
Yes	1.00			
No	15.22 *	1.68–137.89	-	-
Community leader support				
Yes	1.00			
No	4.09 **	1.82–9.17	-	-
Religious leader support				
Yes	1.00			
No	3.84 **	1.78–8.29	-	-
Recommendation from Thai individuals				
Yes	1.00			
No	3.60 **	2.22–5.84	-	-
Information from migrant community				
Yes	1.00			
No	12.04 **	3.18–45.46	-	-
Institutional				
Place of immunization				
Primary health center	1.00			
Government hospital	1.22	0.75–1.99	-	-
Others	5.68 **	1.98–16.26	-	-
Travel distance to clinic (minutes)				
<15	1.00			
≥15	1.84 *	1.12–3.07	-	-
Time taken for one visit (hour)				
<1.5	1.00			
≥1.5	2.12 *	1.32–3.40	-	-
Cost for one visit (THB)				
<150	1.00			
≥150	3.13 **	1.93–5.07	-	-
Reminder from clinic				
Yes	1.00			
No	3.13	0.94–10.46	-	-
Information about AEFIs				
Yes	1.00			
No	3.78 **	2.28–6.27	-	-
Information about schedule				
Yes	1.00			
No	5.09 *	1.84–14.08	-	-
Barriers for immunization				
No	1.00			
Yes	3.79 **	2.34–6.16	-	-
Financial barrier				
No	1.00		1.00	
Yes	9.88 **	3.68–26.53	7.07 *	1.67–29.91
Time barrier				
No	1.00		1.00	
Yes	2.80 *	1.20–6.54	4.09 *	1.20–13.94
Language barrier				
No	1.00			
Yes	3.48 **	2.15–5.64	-	-

* Significant level at α < 0.05. ** Significant level at α < 0.001.

## Data Availability

The original contributions presented in this study are included in the article/Supplementary Materials. Further inquiries can be directed to the corresponding author.

## Supplementary Materials

The following supporting information can be downloaded at: https://www.mdpi.com/article/10.3390/medsci14040452/s1, questionnaire for childhood immunization.

## Abbreviations

The following abbreviations are used in this manuscript:

AEFI	Adverse Events Following Immunization
BCG	Bacille-Camitte-Guerin
BeSD	Behavioral and Social Drivers
COVID-19	Coronavirus disease 19
CSO	Civil Society Organization
DTP	Diphtheria-Tetanus-Pertussis (whole cell)
DTP-HB-Hib	Diphtheria-Tetanus-Pertussis-Hepatitis B-Hemophilus influenza vaccine
EPI	Expanded Program on Immunization
HB	Hepatitis B vaccine
IPV	Inactivated Polio Vaccine
LAJE	Live Attenuated Japanese Encephalitis Vaccine
LMICs	Low- and Middle-Income Countries
M-Fund	Migrant Fund
MHIS	Migrant Health Insurance Scheme
MHV	Migrant Health Volunteer
MMR	Mumps-Measles-Rubella
NGO	Non-Government Organization
OPV	Oral polio vaccine
PACV	Parental Attitudes about Childhood Vaccines
Rota	Rota virus
SEA	Southeast Asia
SEZ	Special Economic Zone
UNICEF	United Nations Children’s Fund
WHO	World Health Organization
