# Supplementary material for: Factors Associated with Immunization Status Among Myanmar Migrant Children in Northern Thailand: A Community-Based Cross-Sectional Study
Source: Med Sci (Basel). 2026 Aug 2;14(4):452. doi: 10.3390/medsci14040452 (PMC13515268; doi:10.3390/medsci14040452)
Supplement: Supplementary file 1 [file medsci-14-00452-s001.zip › medsci-4428976-supplementary.pdf]

QUESTIONNAIRE FOR CHILDHOOD IMMUNIZATION

The survey is to be completed by parents or caregivers of children under age 7 years.

Date …………………………….

Research code ………………….

District name……………….......

Part 1: Socio-demographic Characteristics Questionnaires

Please read the following questions carefully and choose the most appropriate answers in your opinion.

For child

1. Age of child: ________ months/ years

2. Sex of child
   ☐ Boy ☐ Girl

3. Educational status of the child
   ☐ Not schooling yet ☐ Preschool/ kindergarten ☐ Primary school

4. Country of birth
   ☐ Thailand ☐ Myanmar

5. Birth setting
   ☐ Home-based ☐ Health facility-based

6. If your child was born in Thailand, is he/she officially registered at the local civil registration office (District Office or Amphoe)?
   ☐ Yes ☐ No

7. What kind of health insurance does your child have?
   ☐ Don't have ☐ Migrant health insurance scheme
   ☐ Migrant fund (M-fund) ☐ Others (please specify): ……………………………….

8. How many times did mother receive antenatal care for this child?
   ☐ < 5 times ☐ > / = 5 times ☐ No AN care

For caregivers

9. Relationship to the child
   ☐ Mother ☐ Father ☐ Grandparents ☐ Uncle or aunt ☐ Brother or sister
   ☐ Other (please specify): …………………….

10. Sex
   ☐ Male ☐ Female

11. Ethnicity
    ☐ Bamar ☐ Karen ☐ Shan ☐ Mon ☐ Others……….

12. What is your religion?
    ☐ Buddhist ☐ Christian ☐ Islam ☐ Others………………

13. Highest educational attainment of caregiver:
    ☐ No formal education ☐ Primary school ☐ Middle school ☐ High school
    ☐ More than high school

14. Occupation:
    ☐ Unemployed ☐ Agriculture and livestock ☐ Trader ☐ Service provider
    ☐ Construction worker ☐ Others (please specify): …………………………..

15. How long have you been in this community?

    ______ Years ______ Months

16. Family income per month: __________ Baht

17. Total number of children: __________ children

18. In what permission status are you staying in Thailand?
    ☐ Documented ☐ Undocumented

Part 2: Assessment of child immunization status

1. Do you have vaccination card with appointment?

   ☐ Yes (Record immunization date from vaccination card)
   ☐ No/ Don't know/ Lost/ No response

2. Immunization status
   ☐ Fully ☐ Partially ☐ non-immunized

3. If incomplete, why was the child not completely immunized? (multiple answers)
   ☐ I move from place to place often, which makes it hard to complete vaccines.

☐ I often forget dates of vaccination appointments for my child.
☐ I worry about vaccine side effects, such as fever, for my child
☐ I do not have enough time to take the child for vaccination because of work.
☐ Others (please specify): ……………………………

Part 3: Knowledge for immunization questionnaires

This questionnaire is designed to assess your knowledge about childhood immunization. Please read each question carefully and answer your honest responses. Put a true mark in only one place.

No.	Statements	Yes	No	Don't know/ no response
1.	Immunization can prevent childhood vaccine preventable diseases.			
2.	The child must be started immunization since birth or as early as possible.			
3.	Hepatitis B vaccine can prevent your child from Hepatitis B viral infection.			
4.	Pertussis or whooping cough disease cannot be prevented by vaccine.			
5.	Measles is a vaccine preventable disease.			
6.	Vaccine for TB (BCG) and the first dose of Hepatitis B (HBV) should be given at birth.			
7.	The first dose of Measles/MMR vaccine should be given after 1 year of age.			
8.	Children need only one dose of polio vaccine before the age of 1 year.			
9.	Mild fever is a common side effect after receiving immunization.			
10.	Soreness or swelling at the site of injection is a common side effect after immunization.			
11.	Diarrhea is a common side effect after immunization.			
12.	Some vaccines can be skipped or delayed if many vaccines are scheduled on the same day.			
13.	If a child has a high fever, the caregiver should consult a health worker before vaccination.			

Part 4: Assessment of attitude on immunization

This assessment asks you how you think, assume, or believe about childhood immunization. Your responses will help us understand caregivers' perspectives on the importance, safety, and effectiveness of vaccines. Please read each statement carefully and respond honestly based on your personal beliefs and experiences. Please choose only one that appears most appropriate.

No.	Statements	Strongly agree	Agree	Not sure	Disagree	Strongly disagree
1.	I believe it is important for children to receive all the recommended shots.					
2.	It is better for my child to get fewer vaccines at the same time.					
3.	Children get more shots than routine requirements are good for them.					
4.	I believe that many of the illnesses that shots prevent are severe.					
5.	It is better for my child to develop immunity by getting sick than to get a shot.					
6.	I trust the information I receive from healthcare providers about shots.					
7.	I am able to openly discuss my concerns about shots with my child's doctor.					

No.	Statements	Not at all concerned	Not too concerned	Not sure	Somewhat concerned	Very concerned
8.	How concerned are you that your child might have a serious side effect from a shot?					
9	How concerned are you that a shot might not prevent the disease?					
No.	Statements	Not at all hesitant	Not too hesitant	Not sure	Somewhat hesitant	Very hesitant
10.	Overall, how hesitant about childhood shots would you consider yourself to be?					

11. On a scale of 1 to 10, how sure are you that following the recommended shot schedule is a good idea for your child?

Not sure at all   Completely sure

0 1 2 3 4 5 6 7 8 9 10

Part 5: Assessment of social or interpersonal drivers for immunization

1. Do you think most of the parents you know get their children vaccinated?
   ☐ Yes ☐ No ☐ Don't know
2. Do you think most of your close family and friends want you to get your child immunized?
   ☐ Yes ☐ No ☐ Don't know
3. Do you think most of your community leaders want you to get your child immunized e.g., village headman, migrant community leader?
   ☐ Yes ☐ No ☐ Don't know
4. Do you think your religious leaders want you to get your child vaccinated?
   ☐ Yes ☐ No ☐ Don't know
5. Has a health care worker recommended your child be vaccinated?

☐ Yes ☐ No ☐ Don’t know

6. How do you receive information about child immunization? (multiple answers)
   ☐ From family members or friends ☐ From community meetings
   ☐ From healthcare providers ☐ From school or daycare centers
   ☐ From social media ☐ Others (please specify): …………………………
7. Do migrant or community health volunteers help you in immunization of your child?
   ☐ Yes ☐ No ☐ Don’t know
8. Have your Thai friends (e.g., employers, landlords, etc.) encouraged you to vaccinate your child?
   ☐ Yes ☐ No ☐ Don’t know
9. If it is time for your children to get vaccinated, would you need permission to take your child to clinic?
   ☐ Yes ☐ No ☐ Don’t know

Part 6: Assessment of health system influence or institutional drivers of immunization

1. Where do you go and get your child immunized?
   ☐ Government hospital
   ☐ Health promotion hospital or district health center
   ☐ Private hospital or clinic
   ☐ School immunization program
   ☐ Others (please specify): ……………………………
2. Approximately distance to health facility for immunization.
   ……………………. km
3. How much time did you spend for the immunization session in general?
   ……………………….. mins or hours
4. How much does it usually cost for one immunization visit for your child? (about the cost, please consider any payments to the clinic, the cost of getting there, plus the cost of taking time away from work)
   ☐ ………………. baht/ visit
   ☐ no cost

5. How easy is it to get immunization services for your child? (choose only one)
   - [ ] Not at all easy - [ ] A little easy - [ ] Moderately easy - [ ] Very easy

6. How easy is it to pay for vaccination? (only one answer)
   - [ ] Not easy at all - [ ] A little easy - [ ] Moderately easy - [ ] Very easy

7. What makes it difficult to get immunization services for your child? (multiple answers)
   - [ ] Nothing (skip the rest)
   - [ ] Getting to the clinic is hard
   - [ ] Financial difficulty
   - [ ] Time constraints
   - [ ] Waiting time in clinic too long
   - [ ] My documentation status creates difficulties
   - [ ] Other challenges you face in getting your child vaccinated (please describe):

   …………………………..

Provider practice

8. Have you ever been contacted (e.g., by phone, text message, home visit) about your child being due for vaccination?
   - [ ] Yes - [ ] No

9. Are there any language barriers when communicating with healthcare providers during immunization appointments?
   - [ ] Yes - [ ] No

10. Is there any interpreter service available during immunization?
    - [ ] Yes - [ ] No - [ ] Don’t know

11. Was your child's body temperature screening conducted before immunization?
    - [ ] Yes - [ ] No - [ ] Don’t know

12. During your child’s last immunization appointment, was there any health message about immunization provided?
    - [ ] Yes - [ ] No - [ ] Don’t know

13. If yes, what is the information about? (multiple answers)
    - [ ] Protection against diseases - [ ] Side effect of vaccination - [ ] Vaccination schedule
    - [ ] Consequences of non-immunization - [ ] Others (please specify): ……………………

Satisfaction

14. How satisfied are you with the immunization services? (only one answer)
    - ☐ Not satisfied at all ☐ A little satisfied
    - ☐ Moderately satisfied ☐ Very satisfied

15. If you are not satisfied, what is not satisfactory about the immunization services?
    - ☐ Providers are poorly trained ☐ The clinic dose not open on time
    - ☐ Waiting time are long ☐ Vaccine is not always available
    - ☐ Providers are not respectful ☐ Other (specify): ……………………………….

Do you have any suggestion or opinion on immunization?

………………………………………………………………………………

………………………………………………………………………………

………………………………………………………………………………

………………………………………………………………………………

Thank you so much for your participation!
